# TIMP-1 mediates TGF-β-dependent crosstalk between hepatic stellate and cancer cells via FAK signaling

**DOI:** 10.1038/srep16492

**Published:** 2015-11-09

**Authors:** Sang-A Park, Min-Jin Kim, So-Yeon Park, Jung-Shin Kim, Woosung Lim, Jeong-Seok Nam, Yhun Yhong Sheen

**Affiliations:** 1 College of Pharmacy, Ewha Womans University, Seoul, South Korea; 2 Department of Surgery, Ewha Womans University School of Medicine, Seoul, South Korea; 3 Laboratory of Tumor Suppressor, Lee Gil Ya Cancer and Diabetes Institute, Gachon University, Incheon, South Korea; 4 Department of Molecular Medicine, School of Medicine, Gachon University, Incheon, South Korea

## Abstract

Transforming growth factor-β (TGF-β) signaling plays a key role in progression and metastasis of HCC. This study was undertaken to gain the proof of concept of a small-molecule inhibitor of TGF-β type I receptor kinase, EW-7197 as a potent anti-cancer therapy for HCC. We identified tissue inhibitors of metalloproteinases-1 (TIMP-1) as one of the secreted proteins of hepatic stellate cells (HSCs) and a key mediator of TGF-β-mediated crosstalk between HSCs and HCC cells. TGF-β signaling led to increased expression of TIMP-1, which activates focal adhesion kinase (FAK) signaling via its interaction with CD63. Inhibition of TGF-β signaling using EW-7197 significantly attenuated the progression and intrahepatic metastasis of HCC in an SK-HEP1-Luc orthotopic-xenograft mouse model. In addition, EW-7197 inhibited TGF-β-stimulated TIMP-1 secretion by HSCs as well as the TIMP-1-induced proliferation, motility, and survival of HCC cells. Further, EW-7197 interrupted TGF-β-mediated epithelial-to-mesenchymal transition and Akt signaling, leading to significant reductions in the motility and anchorage-independent growth of HCC cells. In conclusion, we found that TIMP-1 mediates TGF-β-regulated crosstalk between HSCs and HCC cells via FAK signaling. In addition, EW-7197 demonstrates potent *in vivo* anti-cancer therapeutic activity and may be a potential new anti-cancer drug of choice to treat patients with liver cancer.

Hepatocellular carcinoma (HCC), which is the most frequent type of primary liver cancer, represents the third leading cause of death globally^1^,^2^,^3^,^4^. Although the main cause of death in HCC patients is tumor progression combined with metastasis, the underlying mechanisms of tumor initiation, progression and metastasis are still not fully understood. The increased prevalence of HCC and the lack of effective therapies demand a better understanding of the biology of its progression.

Previous studies have suggested that transforming growth factor-β (TGF-β) may play an important role in the progression of HCC^5^,^6^. In patients with HCC, the TGF-β1 level is correlated with progression and metastasis^7^,^8^,^9^,^10^. TGF-β also induces epithelial-to-mesenchymal transition (EMT), which triggers cell migration and tumor cell invasion in both Smad-dependent and independent manners^6^,^11^. In addition, TGF-β activates hepatic stellate cells (HSCs), which are responsible for the production of cytokines, chemokines, growth factors and an extensive extracellular matrix (ECM)^12^. Activated HSCs infiltrate the stroma of liver tumors and localize around tumor sinusoids, fibrous septa and capsules^13^,^14^. Crosstalk between tumor cells and their surrounding microenvironments plays a central role in the pathogenesis of HCC^5^,^15^. In particular, interactions between HSCs and HCC have been shown to promote the growth and metastasis of HCC^16^. Paracrine and autocrine mechanisms are responsible for crosstalk between cancer cells and surrounding cells^5^,^17^. Targeting of interactions between tumors cells and their microenvironments has emerged as a promising therapeutic strategy. However, the molecular mechanisms that underlie this crosstalk in a tissue-specific context as well as its effects on carcinogenesis remain elusive. One study has reported that TGF-β blockade inhibits the expression of connective tissue growth factor (CTGF) and simultaneously inhibits tumor-stroma crosstalk and tumor progression in HCC^18^. To determine the TGF-β-regulated molecular link between HSCs and HCC, we screened for candidate factors secreted from activated HSCs. We identified tissue inhibitor of metalloproteinases-1 (TIMP-1) as a potent protein secreted by HSCs that advances the progression and metastasis of HCC. Previous studies have reported that TIMP-1 regulates cell proliferation, migration, and survival through its interactions with CD63 on cell surfaces^19^. The binding of TIMP-1 to CD63 activates the focal adhesion kinase (FAK) and phosphoinositide 3-kinase (PI3K) signal transduction pathway, which are important for TIMP-1-mediated cell proliferation, migration, and survival in various cell types^20^,^21^. We found that the disruption of TIMP-1 markedly inhibited the proliferation, migration, and survival of HCC cells and that the silencing of CD63, a specific receptor of TIMP-1, specifically attenuated the TIMP-1-mediated proliferation, migration, and survival of these cells. We further demonstrated that TIMP-1 mediated TGF-β-regulated crosstalk between HSCs and HCC cells through FAK signaling. Based on these data, TGF-β signaling is a potential target for the treatment of HCC, and the direct inhibition of TGF-β signaling has been demonstrated to have therapeutic effects on HCC both *in vitro* and *in vivo*^18^,^22^,^23^,^24^. Recently, we have reported that EW-7197 selectively blocks the kinase activity of TGF-β type I receptor^25^. In this study, we show that EW-7197 inhibits TGF-β signaling to exert therapeutic effects on HCC progression.

## Results

### Inhibition of the Progression of HCC *In Vivo* by Blockade of TGF-β

**Figure 1:**
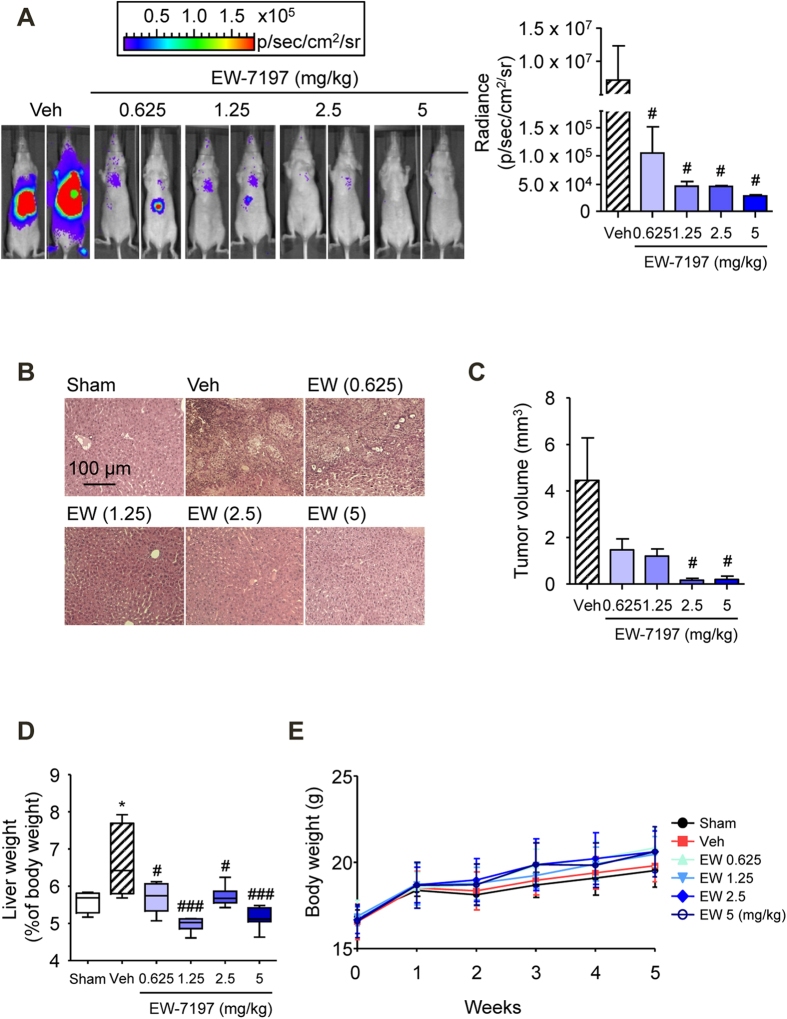
Inhibition of the Progression of HCC *In Vivo* by TGF-β Blockade. (**A**) Effects of EW-7197 (EW) on HCC progression in HCC mice. The sizes of liver tumors were visualized by bioluminescence analysis. The image (left) shows the tumor sizes in livers of two representative mice from each group. The plot (right) represents the quantification of bioluminescence intensity as the total flux (photons/second). (**B**) H&E staining of liver tissues from HCC mice. Scale bars: 100 μm. (**C**) Effects of EW-7197 on tumor sizes in HCC mice. (**D**) Effects of EW-7197 on liver weights in HCC mice. (**E**) Effects of EW-7197 on the body weights of HCC mice. ^*^p < 0.05 vs. Sham, ^#^*p* < 0.05 vs. Vehicle, and ^###^*p* < 0.001 vs. Vehicle.

The *in vivo* anti-cancer activity of the TGF-β type I receptor kinase (also called ALK5) inhibitor, EW-7197, was examined in an SK-HEP1-Luc orthotopic-xenograft mouse model of HCC. Athymic nude mice with HCC were treated orally for 21 days with EW-7197 (0.625, 1.25, 2.5, or 5 mg/kg, *qd*). As shown in Fig. 1A, EW-7197 inhibited the growth of hepatic tumors in the mice based on bioluminescence signals in a dose-dependent manner. In addition, the treatment with EW-7197 significantly reduced the volume of the implanted tumors (Figs. 1B,C). EW-7197 blocked the increased Smad2/3 phosphorylation in a dose-dependent manner in the livers and PBMCs of these mice (Supplementary Figs. S1A and S1B). Taken together, ALK5 inhibition by EW-7197 efficiently alleviates HCC *in vivo*. To assess the anti-metastatic activity of EW-7197, we macroscopically counted the number of tumor nodules that were generated on the surface of the livers. EW-7197 at concentration of 0.625, 1.25, 2.5, and 5 mg/kg decreased the numbers of intrahepatic metastatic nodules by 30, 20, 40, and 0%, respectively, compared with the vehicle-treated controls (Table 1). These results suggest that EW-7197 inhibits the intrahepatic metastasis of HCC *in vivo*. Moreover, EW-7197 improved the increased liver weights (Fig. 1D) without causing a concomitant, statistically significant difference in body weights (Fig. 1E). These data show that EW-7197 inhibits HCC progression with little toxicity.

### Suppression of TGF-β-Induced EMT and Akt Signaling *In Vitro* by Blockade of TGF-β

**Figure 2:**
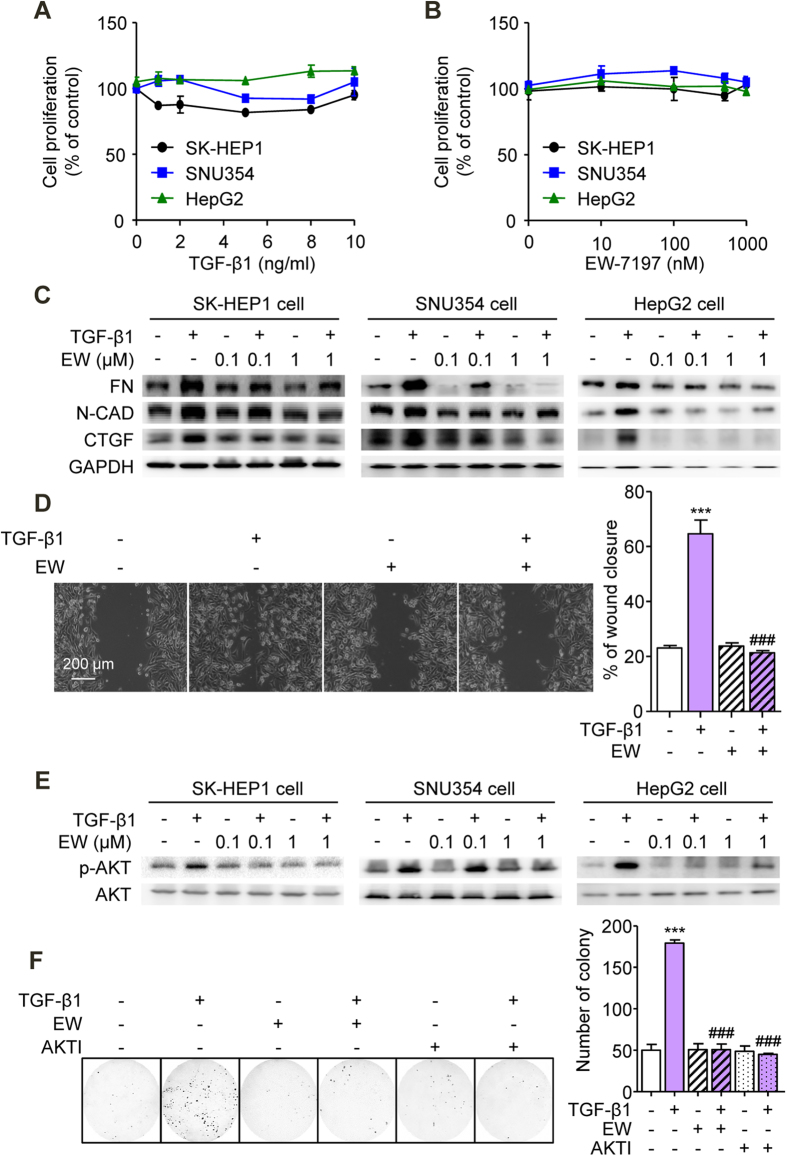
Suppression of TGF-β-Induced EMT and Akt Signaling *In Vitro* by TGF-β Blockade. (**A**) Effects of TGF-β1 on the proliferation of SK-HEP1, SNU354, and HepG2 cells. Cells were treated with the indicated concentration of TGF-β1 for 72 h. (**B**) Effects of EW-7197 on the proliferation of SK-HEP1, SNU354, and HepG2 cells. Cells were treated with the indicated concentration of EW-7197 for 72 h. (**C**) Effects of EW-7197 on the protein expression levels of fibronectin (FN), N-cadherin (N-CAD), and CTGF in SK-HEP1, SNU354, and HepG2 cells. Cells were treated with the indicated concentration of EW-7197 in the presence or absence of TGF-β1 (2 ng/ml) for 24 h. GAPDH was used as a reference. (**D**) Effects of EW-7197 (1 μM) on the migration of SK-HEP1 cells. Cells were treated with EW-7197 in the presence or absence of TGF-β1 (2 ng/ml) for 30 h. Scale bars: 200 μm. (**E**) Effects of EW-7197 on AKT phosphorylation in SK-HEP1, SNU354, and HepG2 cells. Cells were treated with the indicated concentration of EW-7197 in the presence or absence of TGF-β1 (2 ng/ml) for 24 h. AKT was used as a reference. (**F**) Effects of EW-7197 (1 μM) and Akt inhibitor (10 μM, AKTI) on the anchorage-independent growth of SK-HEP1 cells. Cells were treated with the indicated drug in the presence or absence of TGF-β1 (2 ng/ml) for 24 h. After treatment, the cells were counted and placed in soft agar for colony assay to determine cell survival. ^***^p < 0.001 vs. untreated control and ^###^*p* < 0.001 vs. TGF-β1-treated control.

Neither TGF-β1 nor EW-7197 exerted an effect on the proliferation in SK-HEP1, SNU354, or HepG2 cells (Figs. 2A,B). These data indicated that EW-7197 inhibited the growth of HCC cells (Figs. 1A,C) without cytotoxicity. EW-7197 has been previously shown to inhibit ALK5 more strongly than other ALK5 inhibitors in various cell types^26^,^27^. We compared the activity of EW-7197 with those of other ALK5 inhibitors in SK-HEP1-Lux cells using a reporter gene assay. The assessment of TGF-β1-induced luciferase activity indicated that EW-7197 exerted a more potent inhibitory effect than the other ALK5 inhibitors (Supplementary Fig. S2A). Immunofluorescence and Western blot analysis showed that EW-7197 inhibited the TGF-β1-induced phosphorylation of Smad3 in SK-HEP1, SNU354, HepG2, and Huh7 cells *in vitro* (Supplementary Figs. S2B and S2C). Previous studies have reported central roles of Smad proteins in the mediation of TGF-β-induced EMT and have demonstrated their important roles in the progression and intrahepatic metastasis of HCC^6^,^28^. TGF-β1 promotes EMT and induces the up-regulation of mesenchymal markers, such as N-cadherin, fibronectin, and CTGF^28^,^29^. To investigate the inhibitory effects of EW-7197 on mesenchymal markers, we analyzed their protein levels. EW-7197 inhibited the elevations in the protein levels of fibronectin, N-cadherin, and CTGF in SK-HEP1, SNU354, HepG2, and Huh7 cells (Fig. 2C and Supplementary Fig. S3A). We further investigated whether EW-7197 influences cell migration mediated by TGF-β1. In wound-healing assay, EW-7197 attenuated the TGF-β1-induced migration of SK-HEP1 and HepG2 cells (Fig. 2D and Supplementary Fig. S3B). In the non-canonical Smad pathway, TGF-β activates PI3K/Akt signaling, which promotes cell survival^30^. EW-7197 blocked the increased phosphorylation of Akt in a dose-dependent manner in SK-HEP1, SNU354, HepG2, and Huh7 cells (Fig. 2E and Supplementary S3C). To assess the role of Akt signaling in the response of HCC cells to TGF-β, we investigated anchorage-independent cell growth. The TGF-β1-induced anchorage-independent growth of SK-HEP1 cells, as measured as the number of colonies that formed in soft agar assay, was inhibited by EW-7197 and Akt inhibitor V (Fig. 2F). Taken together, these results support the notion that EW-7197 inhibits the progression and intrahepatic metastasis of HCC via the suppression of TGF-β-mediated EMT and Akt signaling.

### Conditioned Media (CM) of TGF-β1-Activated HSCs Increase the Proliferation, Migration and Colony Formation of HCC Cells

**Figure 3:**
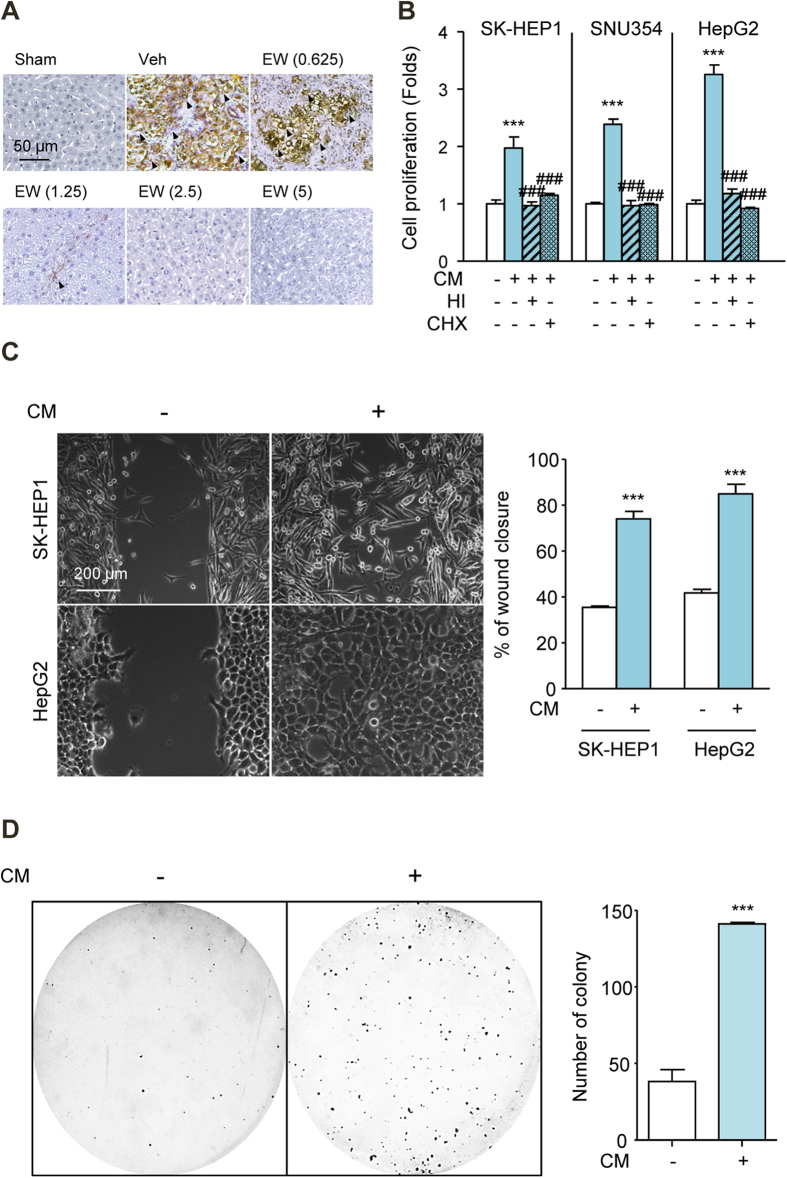
CM from HSCs Increase Proliferation, Migration and Colony Formation of HCC Cells. (**A**) IHC staining for α-SMA in HCC mice. The arrows indicate α-SMA-positive myofibroblasts of liver tissues in HCC mice. Scale bars: 50 μm. (**B**) Effects of conditioned media (CM) from TGF-β activated LX-2 cells on the proliferation of SK-HEP1, SNU354, and HepG2 cells. Cells were treated with CM for 72 h. HI, heat-inactivated; CHX, cycloheximide (10 μg/ml). (**C**) Effects of CM on the migration of SK-HEP1 and HepG2 cells. Cells were treated with CM for 30 h. Scale bars: 200 μm. (**D**) Effects of CM the anchorage-independent growth of SK-HEP1 cells. Cells were treated with CM for 24 h. After treatment, the cells were counted and placed in soft agar for colony assay to determine cell survival. ^***^p < 0.001 vs. untreated control and ^###^*p* < 0.001 vs. CM-treated control.

The communication of tumor cells and stromal cells is known to modulate HCC progression. In particular, the activation of HSC and HSC-HCC cell crosstalk facilitate the progression of HCC^5^,^15^. Because the appearance of α-smooth muscle actin (α-SMA)-positive myofibroblasts is considered a hallmark of HSC activation, we quantified α-SMA expression in HCC-mouse livers. EW-7197 reduced α-SMA levels in the HCC livers, as determined by IHC staining intensity (Fig. 3A). To evaluate the effects of HSCs on the proliferation of HCC cells, HCC cells were exposed to CM from TGF-β1-activated LX-2 cells. This CM increased the proliferation of SK-HEP1, SNU354, and HepG2 cells. These effects were inhibited by either heat-inactivated CM or CM from cycloheximide-treated LX-2 cells (Fig. 3B). These results suggested that LX-2 cells synthesized and secreted proteins that affected the proliferation of HCC cells. We next examined the effects of CM on cell migration and anchorage-independent growth and measured the corresponding responses of SK-HEP1 and HepG2 cells in wound-healing and soft agar assays. CM caused significant increases in SK-HEP1 and HepG2 cells motility (Fig. 3C). In addition, it increased the anchorage-independent growth of SK-HEP1 cells (Fig. 3D). These results confirmed that HSCs promoted increases in the growth and motility of HCC cells *in vitro*.

### Inhibition of Crosstalk between HSC and HCC Cells via the Suppression of TIMP-1 Secretion by TGF-β Blockade

**Figure 4:**
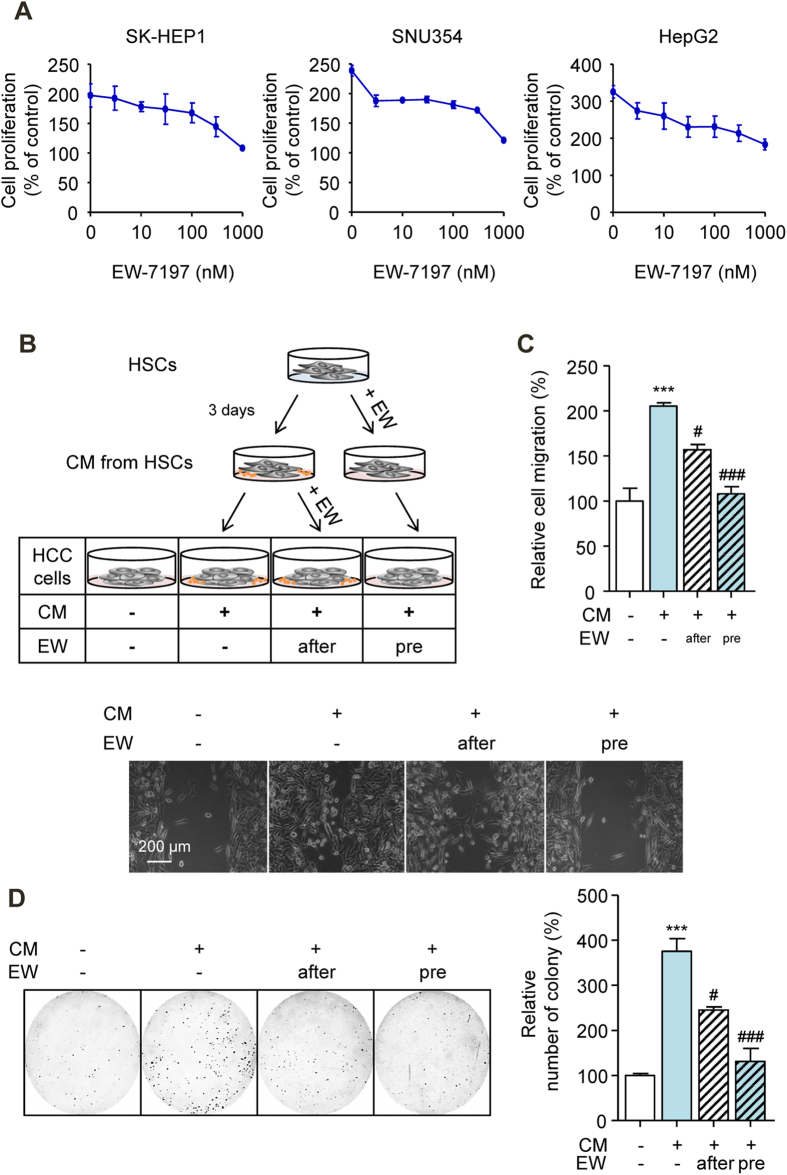
Inhibition of Crosstalk between HSC and HCC Cells via the Suppression of TIMP-1 Secretion by TGF-β Blockade. (**A**) Effects of EW-7197 on the CM-induced proliferation of SK-HEP1, SNU354, and HepG2 cells. Cells were treated with the indicated concentration of EW-7197 in CM for 72 h. (**B**) A schematic for the identification of the effects of EW-7197 on CM-treated cells. (**C**) Densitometric analysis (up) and representative images (down) of wound-healing assay of SK-HEP1 cells. Cells were treated with CM in the presence or absence of EW-7197 (1 μM) for 30 h. Scale bars: 200 μm. (**D**) Representative images (left) and densitometric analysis (right) of soft agar assay of SK-HEP1 cells. Cells were treated with CM in the presence or absence of EW-7197 (1 μM) for 24 h. After treatment, the cells were counted and placed in soft agar for colony assay to determine cell survival. ^***^p < 0.001 vs. untreated control, ^#^*p* < 0.05 vs. CM-treated control, and ^###^*p* < 0.001 vs. CM-treated control.

We then further investigated the effect of EW-7197 on CM from TGF-β1-activated LX-2 cells. CM from EW-7197-treated LX-2 cells reduced CM-induced cell proliferation in a dose-dependent manner in SK-HEP1, SNU354, and HepG2 cells (Fig. 4A). To determine whether EW-7197 acts directly on HCC cells, we cultured HCC cells with CM from LX-2 cells and simultaneously treated them with EW-7197 (after-treatment) (Fig. 4B). CM from EW-7197-treated LX-2 cells (pre-treatment) suppressed the CM-induced migration and survival of SK-HEP1 cells more effectively than after-treatment of EW-7197 (Fig. 4C,D). TGF-β is secreted by HSCs which are transformed to active forms in response to injury^12^. We confirmed that LX-2 cells induced the Smad2/3 phosphorylation and EMT of SK-HEP1 cells in co-culture system (Supplementary Figs. S4A and S4B), indicating paracrine effect of TGF-β. However, we excluded the possibility of the paracrine TGF-β in CM-induced effects *in vitro* experiments due to the fact that secreted TGF-β has a short half-life and CM could not induce 3TP-Lux promoter activity in 3TP-Lux stable SK-HEP1 cells (Supplementary Fig. S4C). These results indicated that EW-7197 inhibited the effects of CM both directly and indirectly. We previously performed luciferase reporter assays using SK-HEP1 cells stably transfected with TGF-β-responsive 3TP-Lux plasmid (Supplementary Fig. S2A). These results indicate that EW-7197 inhibited the TGF-β1-induced luciferase activities (IC_50_= 8.85 nM). Here, luciferase activity is solely depends on TGF-β1 so EW-7197 can inhibit luciferase activity completely. However, in CM there might be other growth factors which is not related to TGF-β, thus EW-7197 shows less inhibition on CM-induced effects than TGF-β-responsive 3TP-Lux reporter assay.

**Figure 5:**
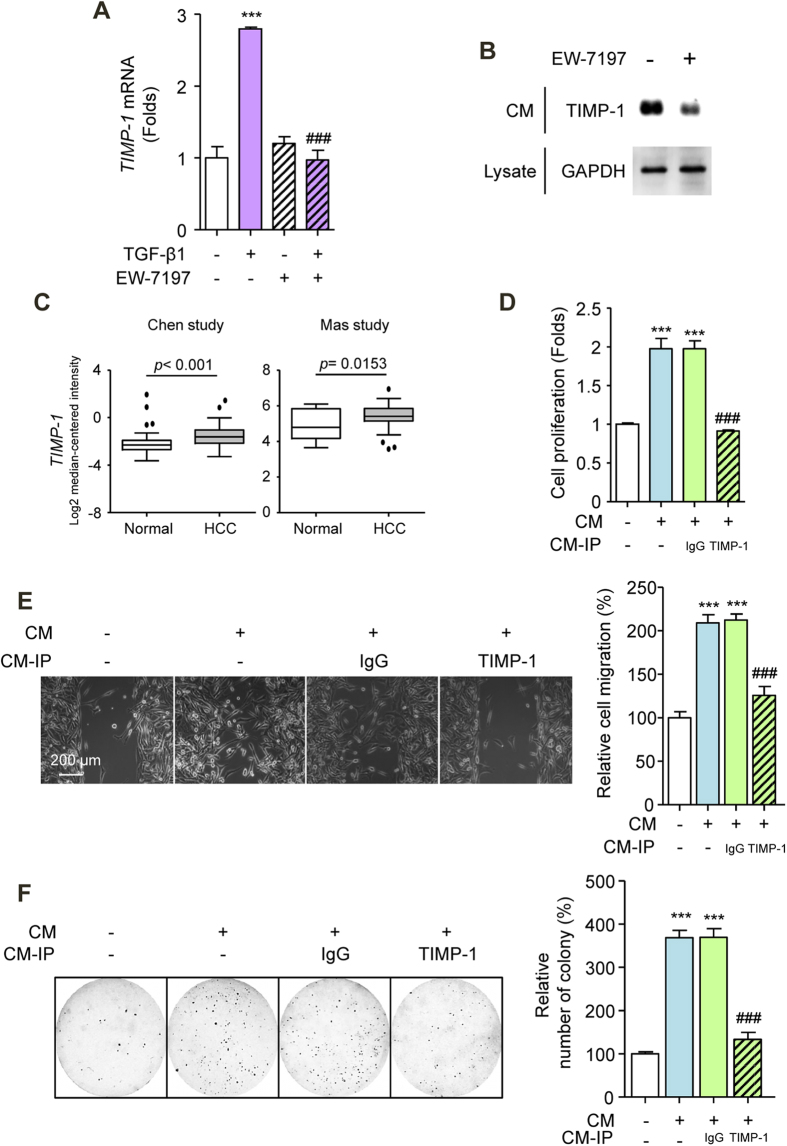
Secreted TIMP-1 Mediates Crosstalk between HSC and HCC Cells. (**A**) TIMP-1 mRNA levels in LX-2 cells. Cells were treated with EW-7197 (1 μM) in the presence or absence of TGF-β1 (2 ng/ml) for 24 h. HPRT was used as a reference. (**B**) TIMP-1 protein levels in CM. The GADPH level in an aliquot of total cell lysate was used as a reference. (**C**) mRNA expression levels of TIMP-1 in HCC patients from the Chen and Mas dataset from Oncomine (www.oncomine.com). (**D**) Effects of TIMP-1 on the proliferation of SK-HEP1 cells. Cells were treated with CM for 72 h. (**E**) Representative images (left) and densitometric analysis (right) of wound-healing assay of SK-HEP1 cells. Cells were treated with CM for 30 h. Scale bars: 200 μm. (**F**) Representative images (left) and densitometric analysis (right) of soft agar assay of SK-HEP1 cells. Cells were treated with CM for 24 h. After treatment, the cells were counted and placed in soft agar for colony assay to determine cell survival. ^***^p < 0.001 vs. untreated control and ^###^*p* < 0.001 vs. CM-treated control.

To identify the potential molecular link between HSCs and HCC cells, we screened for specific candidate factors that are secreted from activated LX-2 cells. First, we performed qRT-PCR analysis and determined that several secreted factors were differentially expressed, including SDF-1, TIMP-2, and TIMP-1, which were abundantly expressed in TGF-β1-activated HSCs (Supplementary Fig. S5A). To investigate the inhibitory effect of EW-7197 on CM, we assessed the mRNA and protein levels of the three candidate secreted factors in LX-2 cells and in CM, respectively. In TGF-β1-activated LX-2 cells, the up-regulated mRNA expression levels of SDF-1, TIMP-2, and TIMP-1 were reduced by EW-7197 (Fig. 5A, Supplementary Figs. S5B and S5C). EW-7197 decreased the protein expression levels of SDF-1 and TIMP-1 in the CM but increased the level of TIMP-2 (Fig. 5B and Supplementary Fig. S5D). Bioinformatic analyses of clinical studies showed that SDF-1 expression was inconsistent among studies, whereas TIMP-1 expression was consistently increased in HCC patients (Fig. 5C and Supplementary Fig. S5E). Moreover, SDF-1 did not affect the proliferation of SK-HEP1, SNU354, or HepG2 cells (Supplementary Fig. S6). Thus, we then assessed TIMP-1 as factor secreted from TGF-β1-activated LX-2 cells that is involved in crosstalk between HSCs and HCC cells. Depletion of TIMP-1 from CM by immunoprecipitation reduced its proliferative effect on SK-HEP1 cells (Fig. 5D). Consistently, TIMP-1-immunoprecipitated CM suppressed the migration and anchorage-independent growth of SK-HEP1 cells (Figs. 5E,F). These data implicate TIMP-1 as an active factor in CM from TGF-β1-activated LX-2 cells. Furthermore, to determine whether extracellular TIMP-1 is sufficient to promote CM-induced effects, we incubated SK-HEP1 cells with recombinant TIMP-1. We found significant increases in the proliferation, migration, and colony formation of SK-HEP1 cells upon both recombinant human and mouse TIMP-1 stimulation (Supplementary Figs. S7A-S7C). Our results indicate that TIMP-1 of mouse origin is capable of promoting the proliferation, migration, and anchorage-independent growth of human HCC cells that result in progression of HCC *in vivo*.

### Secreted TIMP-1 Activates the FAK Signal Transduction Pathway in HCC Cells

**Figure 6:**
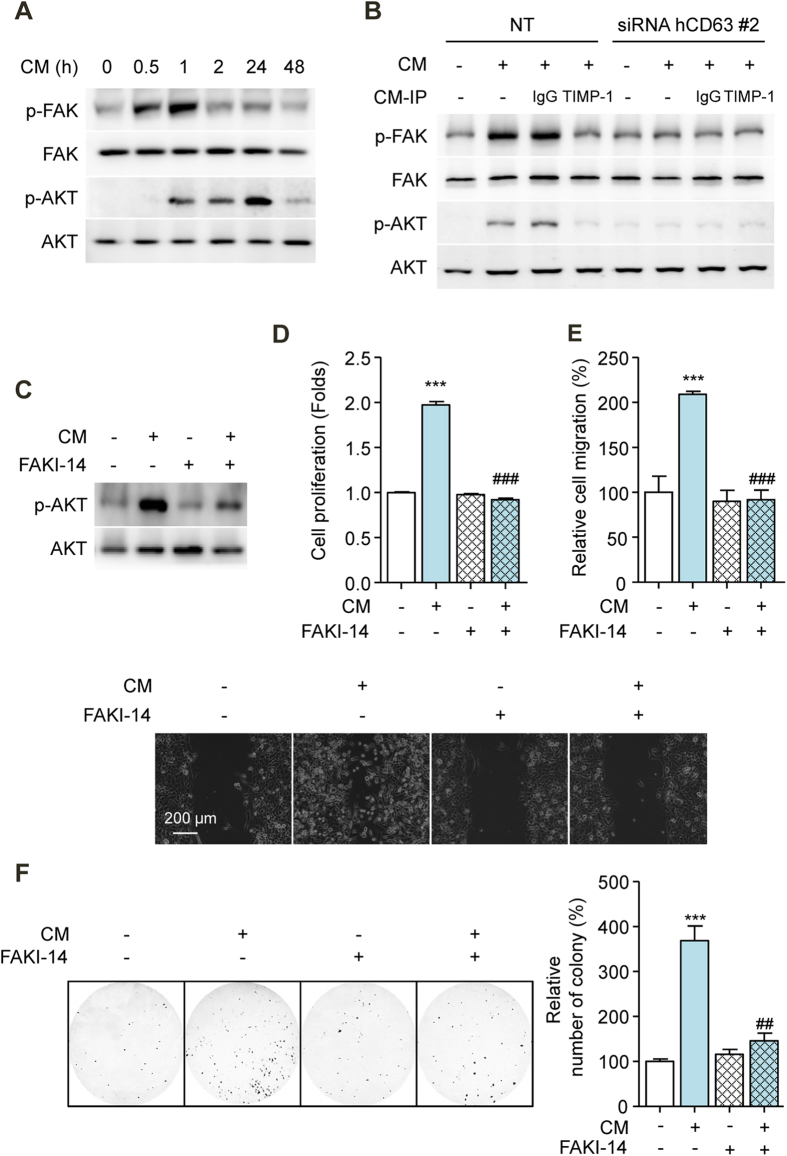
Secreted TIMP-1 Activates the FAK Signal Transduction Pathway in HCC Cells. (**A**) Effects of CM on FAK and AKT phosphorylation in SK-HEP1 cells. FAK and AKT were used as references. (**B**) Effects of CM on FAK and AKT phosphorylation in CD63-siRNA-transfected SK-HEP1 cells. Cells were treated with CM for 1 h (p-FAK and FAK) and 24 h (p-AKT and AKT), respectively. FAK and AKT were used as references. (**C**) Effects of FAK inhibitor-14 (10 μM, FAKI-14) on AKT phosphorylation in SK-HEP1 cells. Cells were treated with CM in the presence or absence of FAK inhibitor-14 for 24 h. AKT was used as a reference. (**D**) Effects of FAK inhibitor-14 on the proliferation of SK-HEP1 cells. Cells were treated with CM in the presence or absence of FAK inhibitor-14 for 72 h. (**E**) Densitometric analysis (up) and representative images (down) of wound-healing assay of SK-HEP1 cells. Cells were treated with CM in the presence or absence of FAK inhibitor-14 for 30 h. Scale bars: 200 μm. (**F**) Representative images (left) and densitometric analysis (right) of soft agar assay of SK-HEP1 cells. Cells were treated with CM in the presence or absence of FAK inhibitor-14 for 24 h. After treatment, the cells were counted and placed in soft agar for colony assay to determine cell survival. ^***^p < 0.001 vs. untreated control, ^##^*p* < 0.01 vs. CM-treated control, and ^###^*p* < 0.001 vs. CM-treated control.

Previous studies have reported that TIMP-1 regulates cell proliferation, migration, and survival through its interactions with CD63 on cell surfaces^19^. Consistent with previous observations, an immune-complex of CD63 with TIMP-1 was detected from CM-treated SK-HEP1, SNU354, and HepG2 cells (Supplementary Figs. S8A and S8B). When TIMP-1 bind to CD63, the FAK and PI3K signal transduction pathways are activated, and this activation is important for TIMP-1-mediated cell proliferation, migration, and survival in various cell types^20^,^21^. We hypothesized that the effect of secreted TIMP-1 on HCC cells may due to its interaction with CD63 on cell surfaces. We first examined the effect of CM on the activation of signal transduction in HCC cells. CM caused a significant increase in FAK and Akt phosphorylation in SK-HEP1 cells (Fig. 6A). To confirm that the interaction between TIMP-1 and CD63 was involved in the activation of signal transduction pathways, a CD63 knockdown experiment using siRNA was performed in SK-HEP1 cells. CD63 gene expression was effectively decreased by CD63 siRNA compared with NT siRNA (Supplementary Figs. S9A and S9B). CM-induced FAK and Akt phosphorylation was almost completely abolished by TIMP-1 immunoprecipitation in SK-HEP1 cells. Additionally, CM did not activate FAK or Akt signal transduction in SK-HEP1 cells that were transfected with CD63 siRNA compared with those that were transfected with NT siRNA (Fig. 6B). These results showed that the interaction between TIMP-1 and CD63 regulated the activation of FAK and Akt signal transduction. Interestingly, EW-7197 treatment reduced the expression of CD63 in SK-HEP1 cells (Supplementary Fig. S9C). These results were relevant to explain the direct inhibition of EW-7197 on CM-induced effects. To determine whether TIMP-1 mediates the proliferation, migration, and survival of HCC cells thorough FAK signaling, we examined the effect of FAKI-14 in CM-treated SK-HEP1 cells. FAKI-14 significantly attenuated the CM-induced phosphorylation of Akt and the proliferation, migration, and anchorage-independent growth of SK-HEP1 cells (Fig. 6C,F). Taken together, these results showed that the interaction of TIMP-1-CD63 mediated the crosstalk between HSCs and HCC cells via the FAK signal transduction pathway.

## Discussion

**Figure 7:**
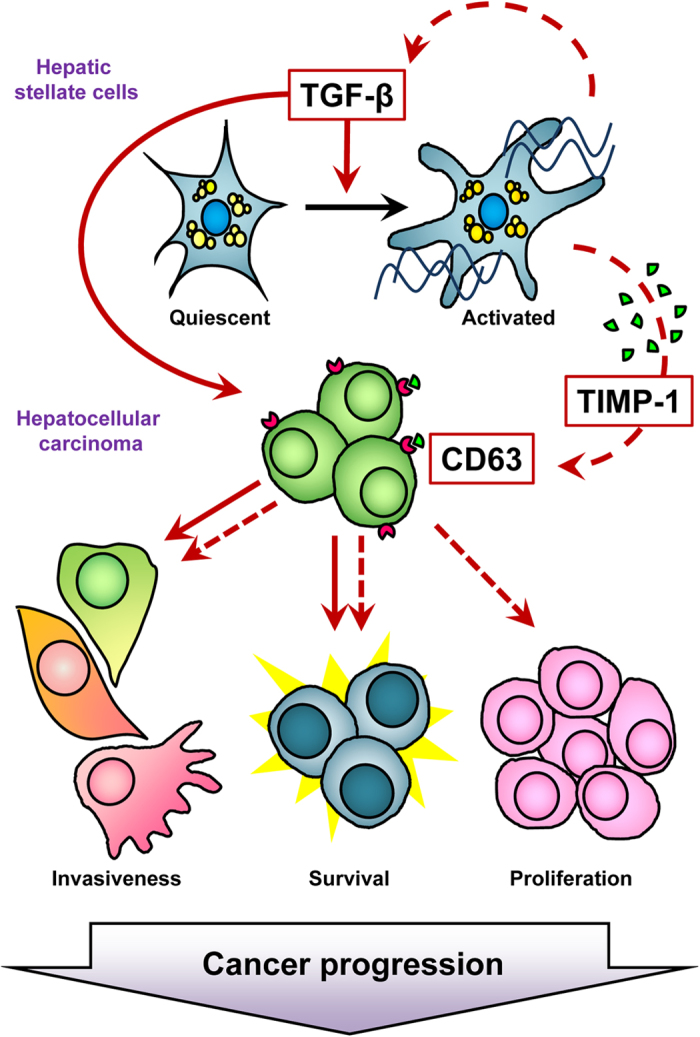
Proposed Model of Action of TGF-β during HCC Progression. Schematic representation of direct (solid line) and indirect (dotted line) action of TGF-β in HCC. TGF-β directly induces cell invasiveness and survival of HCC cells. In addition, TGF-β activates HSCs and increases secretion of TIMP-1 and TGF-β from HSCs. Secreted TIMP-1 leads to increase HCC cells proliferation, invasiveness and survival through binding of TIMP-1 to the TIMP-1 receptor, CD63 expressed on HCC cells.

HCC is the most common type of primary liver cancer, and it typically develops in livers with hepatic fibrosis and cirrhosis^31^. TGF-β plays a crucial role in the molecular pathogenesis of HCC and intervenes in the processes of hepatic fibrosis and cirrhosis^32^. It is also involved in crosstalk between tumors and their microenvironments, notably in activated HSCs, during the onset and progression of HCC^16^. Based on these findings, TGF-β signaling appears to be a potential target for HCC treatment, and the direct inhibition of ALK5 might be an attractive approach to prevent detrimental tumor progression due to TGF-β signaling. In this study, we report for the first time that EW-7197, a selective ALK5 inhibitor, blocks the production of TIMP-1 in TGF-β1-activated HSCs, which consequently interrupts communication between HSCs and HCC cells, and inhibits the progression and intrahepatic metastasis of HCC. This study has demonstrated that tumor-associated HSCs secrete TIMP-1 and affect the proliferation, motility, and anchorage-independent growth of HCC cells (Fig. 7). *In vitro* assays have established that TIMP-1 promotes crosstalk between HSCs and HCC cells through interactions with CD63 and the activation of FAK signaling.

TIMP-1 is already well known as a regulator of matrix metalloproteinase (MMP) activity^33^. Although it was originally characterized by its ability to inhibit MMP activity, it is now widely recognized to have additional biological activities that are independent of MMPs^21^. Several recent studies have reported that high concentration of serum TIMP-1 is associated with worse prognosis in HCC patients and TIMP-1 contributes to HCC progression. Song and colleagues reported that TIMP-1 activates carcinoma-associated fibroblasts and suppresses tumor apoptosis via SDF-1/CXCR4 signaling, which promotes HCC progression^34^. Consistent with previous observations, we provide evidence that TIMP-1 functions as a paracrine mediator in the interaction between HSCs and HCC cells. Additionally, we showed that activated HSCs release SDF-1, which is inhibited by ALK5 inhibitor (Supplementary Figs S5A, S5B, and S5D). These observations point to the underlying mechanisms for effects of ALK5 inhibitor on HCC progression.

As reported previously, TIMP-1 performs its biological activities through interaction with CD63 (a member of the tetraspanin family) on cell surfaces^19^. Consistent with these reports, we have shown that CD63 plays an important role in the TIMP-1-mediated proliferation, migration, and survival of HCC cells. Downstream of CD63, TIMP-1 activates the FAK-Akt pathway to protect a variety of cell types from apoptosis in a manner that is independent of its metalloproteinase inhibitory activity^35^,^36^,^37^,^38^,^39^. However, the mechanism underlying the TIMP-1-CD63-mediated activation of the FAK-Akt pathway in HCC cells remains elusive. Here, we showed that secreted TIMP-1 from activated HSCs potentiated FAK and Akt signaling in HCC cells. In addition, the involvement of FAK signaling in the proliferation, migration, and survival of HCC cells was directly demonstrated by treatment with FAKI-14, which caused the dramatic inhibition of CM-induced effects. These results are consistent with those of previous studies demonstrating that the activation of the FAK signal transduction pathway plays important roles in cell proliferation, motility, and colony formation^19^,^36^,^37^,^38^,^39^,^40^. Our results suggest that TIMP-1-mediated crosstalk between HSCs and HCC cells affects the progression of HCC through the activation of FAK signaling.

FAK signaling is known to control cell movement, invasion, survival, gene expression and cancer stem cell (CSC) self-renewal^41^. Thus, FAK may be a key signaling protein in the control of stem cell proliferation. CSCs or tissue-specific progenitor cells can facilitate tumor growth and FAK signaling has been linked to the maintenance of these cell types. As an alternative approach to test the effects of CM in HCC cells, we evaluated whether it affects the feature of CSCs. Interestingly, CM increased the self-renewal of SK-HEP1, SNU354, and HepG2 cells (Supplementary Fig. S10AM). Flow cytometric analysis revealed an increase in the CD44^+^ CD90^+^ fraction in CM-treated SK-HEP1 cells (Supplementary Fig. S10B). CM increased the mRNA levels of OCT4, NANOG, SOX2, KLF4, and C-MYC in SK-HEP1, SNU354, and HepG2 cells (Supplementary Fig. S10C). To further confirm the effects of CM-induced FAK signaling on CSC features in HCC cells, we inhibited FAK signaling in CM-treated SK-HEP1 cells. This inhibition significantly reduced the number of secondary spheres (Supplementary Fig. S11A) and decreased CD44/CD90 expression in SK-HEP1 cells (Supplementary Fig. S11B). qRT-PCR analysis showed that the inhibition of FAK decreased the mRNA expression of OCT4, NANOG, SOX2, KLF4, and C-MYC in SK-HEP1 cells (Supplementary Fig. S11C). Altogether, these results indicate that FAK signaling regulates the CSC features of HCC cells. Although further understanding of the mechanisms that underlie the role of CSCs in the progression of HCC are required, our study provides proof of concept for the use of ALK5 inhibitors as potential therapeutic agents for HCC that act via the inhibition of FAK activation.

In conclusion, we have found that the targeting of ALK5 yields both direct and indirect therapeutic effects on HCC progression. Our study has demonstrated that the inhibition of TGF-β signaling may be a promising therapeutic strategy because it interferes with the growth of tumors and the interactions of tumors with their microenvironments.

## Materials and Methods

### Reagents

EW-7197, SB-505124, and LY2157299 were synthesized and kindly provided by Dr. DK Kim (Ewha Womans University, Seoul, Korea). Recombinant human TGF-β1 and mouse TIMP-1 were purchased from R&D Systems (Minneapolis, MN, USA). Recombinant human TIMP-1 were purchased from PeproTech (Rocky Hill, NJ, USA). Akt inhibitor V (triciribine) was purchased from Calbiochem (San Diego, CA, USA). Cycloheximide, stromal cell-derived factor 1 (SDF-1), and FAK inhibitor-14 (FAKI-14) were purchased from Sigma-Aldrich (St Louis, MO, USA).

### Cell Culture and Transfection

The immortalized human activated HSC line LX-2, was obtained from Dr. SG Kim (Seoul National University, Seoul, Korea). The human HCC cell lines SK-HEP1 and HepG2 as well as LX-2 cells were maintained in Dulbecco’s modified Eagle’s medium (DMEM) supplemented with 10% fetal bovine serum (FBS). SNU354 and Huh7 human HCC cells were maintained in Roswell Park Memorial Institute media supplemented with 10% FBS. All media were purchased from Gibco Life Technologies (Gaithersburg, MD, USA). Cells were maintained at 37 °C in a humidified incubator in the presence of 5% CO_2_.

SK-HEP1 cells were seeded into 24-well plates, and plasmids were transfected using Tfx™-50 (Promega, Madison, WI, USA) according to the manufacturer’s protocol. SK-HEP1-Luc and 3TP-Lux stable cells were cultured for 4 weeks in the presence of G418 (0.5 mg/ml). Several single clones were isolated, and luciferase activities were measured.

For the silencing of CD63, SK-HEP1 cells were transfected in six-well plates using Lipofectamine 2000 (Invitrogen, Carlsbad, CA, USA) according to the manufacturer’s protocol, with 100 nM antisense CD63 (Bioneer, Daejeon, Korea). On-target plus nontargeting (NT) siRNA (Bioneer) was used as a negative control (Supplementary Table S1). mRNA and protein levels were measured at 24 h after transfection.

### Animals, Treatments and Specimen Collection

Female BALB/c-nu/nu mice were purchased from Central Lab Animal, Inc. (Seoul, Korea). The mice were housed with five to seven animals per cage at room temperature under a 12-h light/dark cycle. A normal chow diet and water were provided ad libitum. All experimental procedures were approved by the Institutional Animal Care and Use Committee of Ewha Womans University and complied with the NIH Guide for the Care and Use of Laboratory Animals (Institute of Laboratory Animal Resources, National Research Council, WA, USA). At 1 h after the last EW-7197 administration, the animals were deeply anaesthetized by intraperitoneal injection of Zoletil/Rompun (2:1 mixture). The animals were exsanguinated via the axillary artery, followed by the collection of sera and excision of the liver, spleen, kidneys, and lungs. The sera and tissues were immediately stored at −80 °C until use.

### Orthotopic–Xenograft HCC Mouse Model

SK-HEP1-Luc cells [2 × 10^6^ cells/20 μl phosphate buffered saline (PBS)] were implanted into the left lobes of the livers of female BALB/c-nu/nu mice (day 0). Their body weights were measured weekly. EW-7197 treatment began when the development of liver tumors was confirmed by autopsy (day 14). EW-7197 was dissolved in an artificial gastric fluid formulation (Veh) and animals were treated with EW-7197 at 0.625, 1.25, 2.5, or 5 mg/kg five times a week for 3 weeks to determine the existence of a possible dose-response relationship^27^. On day 35, the mice were analyzed with an *in vivo* imaging IVIS-200 system (Xenogen Corporation, Hopkinton, MA) to monitor the liver tumor sizes. Captured images were quantified using Living Image Software package (PerkinElmer/Caliper Life Sciences, Hopkinton, MA, USA).

### Preparation of CM from TGF-β1-activated HSCs

To obtain CM from TGF-β1-activated HSCs, LX-2 cells were pretreated with TGF-β (2 ng/ml) for 24 h. After treatment, activated LX-2 cells were seeded in 10-cm cell culture dishes at 3.5 × 10^5^ cells per dish and were then cultured for 3 days in DMEM media supplemented with 0.2% FBS. The CM were harvested and centrifuged with a FLETA5 centrifuge (Hanil Science Industrial Co., Ltd., Incheon, Korea) at 716 g for 3 min to remove cellular debris.

### Immunohistochemistry (IHC)

Tissues were fixed and stained with hematoxylin and eosin. For IHC analysis, deparaffinized sections were washed with PBS and were then treated with 3% hydrogen peroxide for 5 min. The sections were blocked with 10% normal goat serum in Tris-HCl-buffered saline or horse serum in PBS for 1 h and then incubated with primary antibodies for 1 h at room temperature (Supplementary Table S2). After washing, the sections were incubated with the appropriate secondary antibodies (biotin-conjugated IgG; Vector Laboratories, Burlingame, CA, USA) and were then developed with a Vecta-Elite streptavidin-peroxidase kit (Vector Laboratories). The sections were counterstained with diluted hematoxylin and examined by light microscopy (Carl Zeiss, Jena, Germany).

### Immunofluorescence

Cells were fixed in a 4% paraformaldehyde solution (pH 7.4), blocked in 5% bovine serum albumin, and then incubated with primary/secondary antibodies. The cells were then counterstained with 4’6-diamidino-2-phenylindole (DAPI). Images were obtained with an LSM 510 META laser confocal microscopy system (Carl Zeiss, Jena, Germany) at a magnification of 400x.

### Western Blot Analysis

Western blot analysis was performed as previously described^27^. The antibodies used are listed in Supplementary Table S2.

### Reverse Transcription-Polymerase Chain Reaction (RT-PCR) Analyses

Real-time RT-PCR analysis was performed as previously described^27^. The primers used are listed in Supplementary Table S3.

### Immunoprecipitation

The CM were incubated with a TIMP-1 antibody in a rotator at 4 ^o^C for 18 h. Protein A agarose/salmon sperm DNA (Millipore) was added, and rotational incubation was continued for an additional 3 h. Immunoprecipitated CM were collected by centrifugation at 8,000 g at 4 °C for 3 min.

### Cell Counting Kit-8 (CCK-8) Assay

Cell proliferation was measured using a CCK-8 assay kit (Dojindo Laboratories, Kumamoto, Japan). Cells were seeded in 96-well plates and allowed to attach overnight. The cells were then treated with EW-7197 or FAKI-14 (10 μM) in CM for 72 h. After these treatments, CCK-8 solution (10 μl) was added to each well, and the wells were incubated for another 1 h at 37 °C. Absorbance was measured at 450 nm with an ELISA microplate reader (Versa Max; Molecular Devices, Sunnyvale, CA, USA).

### Wound-Healing Assay

Cells were seeded onto 35-mm dishes that contained a cell culture insert (ibidi, Munich, Germany) and allowed to attach overnight. After removal of the cell culture insert, which created a 500-mm gap between the cell patches, the cells were treated with media for 30 h. The wound area was measured at the starting point or at the end point using the Image J program to examine phase-contrast images of cells that were captured with a camera attached to a microscope (Carl Zeiss) at a magnification of 100x. The closure of the wound area was calculated as a percentage of the initial wound area.

### Soft Agar Assay

Soft agar colony formation assay was performed in 6-well plates at a density of 5 × 10^3^ cells per well. Cells suspended in 1.5 ml of top agar (0.35% agar in culture medium) were overlaid onto a layer of 1.5 ml of bottom agar (0.7% agar in the same culture medium). After 14 days, the colonies were photographed and scored by inverted phase-contrast microscopy without fixation or staining.

### Statistical Analyses

The results are expressed as the mean ± the standard error of the mean (SEM) or the standard deviation (SD). Statistical comparisons were determined by one-way analysis of variance followed by Dunnett’s two-tailed post hoc test (SPSS ver. 10.0; SPSS, Chicago, IL, USA). Values of *p* *<* 0.05, 0.01 or 0.001 were considered significant.

## Additional Information

**How to cite this article**: Park, S.-A. *et al.* TIMP-1 mediates TGF-β-dependent crosstalk between hepatic stellate and cancer cells via FAK signaling. *Sci. Rep.* **5**, 16492; doi: 10.1038/srep16492 (2015).

## Supplementary Material

Supplementary Materials

## Figures and Tables

**Table 1:** Intrahepatic Metastasis in a Mouse Model of HCC.

Groups (mg/kg)	Number of mice with macroscopic intrahepatic metastatic nodules (%)
Veh	5/7 (71)
EW (0.625)	3/6 (50)
EW (1.25)	4/7 (57)
EW (2.5)	3/7 (43)
EW (5)	0/6 (0)

EW-7197 (EW: 0.625, 1.25, 2.5, or 5 mg/kg *qd*) dissolved in an artificial gastric fluid formulation (Veh) was given to mice orally five times per week for 3 weeks. The inoculation of tumor cells into the left lobe of the liver was confirmed by histology and H & E staining.
