# Supplementary material for: TIMP-1 mediates TGF-β-dependent crosstalk between hepatic stellate and cancer cells via FAK signaling
Source: Sci Rep. 2015 Nov 9;5:16492. doi: 10.1038/srep16492 (PMC4637930; doi:10.1038/srep16492)
Supplement: Supplementary Materials [file srep16492-s1.pdf]

TIMP-1 mediates TGF-β-dependent crosstalk between hepatic stellate and cancer cells via FAK signaling

Sang-A Park[1], Min-Jin Kim[1], So-Yeon Park[1], Jung-Shin Kim[1], Woosung Lim[2], Jeong-Seok Nam[3, 4, *] & Yhun Yhong Sheen[1, *]

[1]College of Pharmacy, Ewha Womans University, Seoul, South Korea.

[2]Department of Surgery, Ewha Womans University School of Medicine, Seoul, South Korea.

[3]Laboratory of Tumor Suppressor, Lee Gil Ya Cancer and Diabetes Institute, Gachon University, Incheon, South Korea.

[4]Department of Molecular Medicine, School of Medicine, Gachon University, Incheon, South Korea.

[*]**Corresponding author:** YY Sheen, College of Pharmacy, Ewha Womans University, Seodaemun-gu, Seoul, 120-750, Korea. Telephone number: +82-2-3277-3028; fax: +82-2-3277-2851; e-mail: yysheen@ewha.ac.kr and J-S Nam, Laboratory of Tumor Suppressor, Lee Gil Ya Cancer and Diabetes Institute, Gachon University, 7-45 Songdo-dong, Yeonsu-ku, Incheon, 406-840, Korea. Telephone number: +82-32-899-6072; fax: +82-32-899-6350; e-mail: namjs@gachon.ac.kr

Supplementary figures

Supplementary Fig. S1.

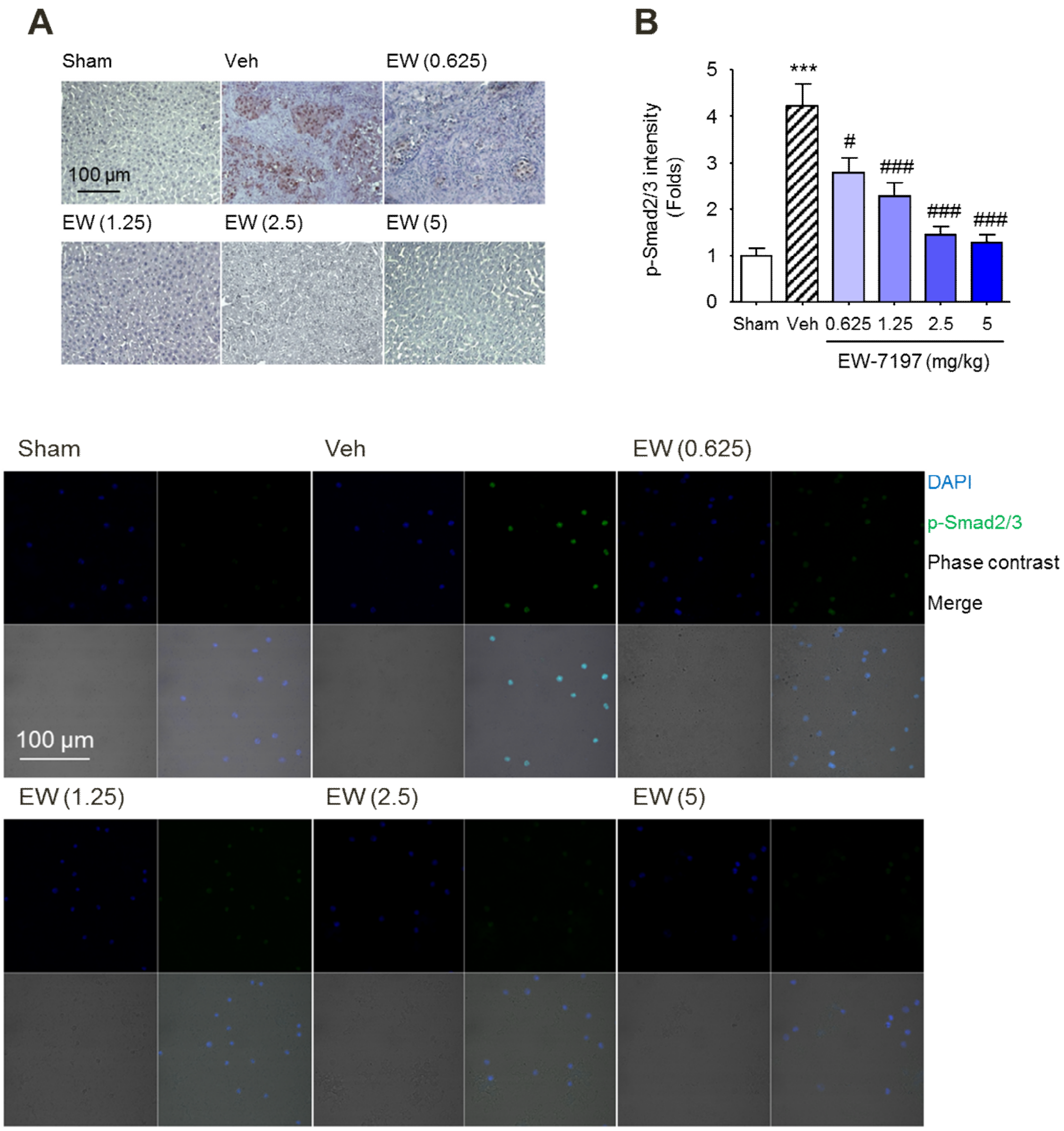

(A) IHC staining for p-Smad2/3 in HCC mice. (B) Densitometric analysis (up) and representative images (down) of p-Smad2/3 expression in PBMCs of HCC mice. Scale bars: 100 μm. $^{***}p<0.001$ vs. Sham, $^{\#}p<0.05$ vs. Vehicle, and $^{\#\#\#}p<0.001$ vs. Vehicle.

Supplementary Fig. S2.

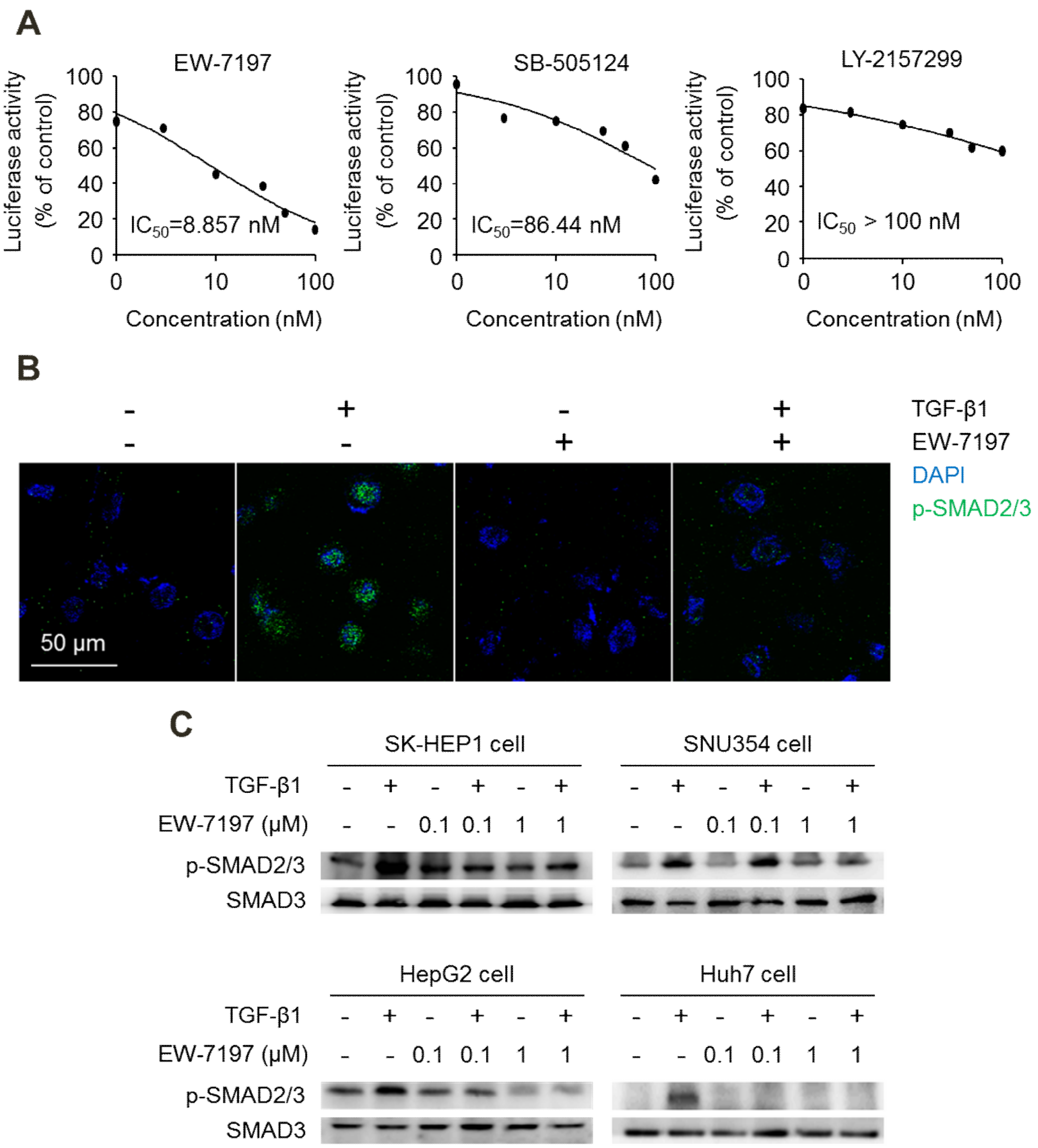

(A) Effects of ALK5 inhibitors on 3TP-Lux promoter activity induced by TGF-β1 in SK-HEP1 3TP-Lux stable cells. Cells were treated with the indicated concentration of drug in the presence of TGF-β1 (2 ng/ml) for 24 h. (B) Effects of EW-7197 (1 μM) on the nuclear translocation of p-Smad2/3 in SK-HEP1 cells. Cells were treated with EW-7197 in the presence or absence of TGF-β1 (2 ng/ml) for 3 h. p-Smad2/3 and nuclei were stained with Alexa Flour 488 (green) and DAPI (blue), respectively. Scale bars: 50 μm. (C) Effects of EW-7197 on Smad2/3 phosphorylation in SK-HEP1, SNU354, HepG2, and Huh7 cells. Cells were treated with the indicated concentration of EW-7197 in the presence or absence of TGF-β1 (2 ng/ml) for 3 h. Smad3 was used as a reference.

Supplementary Fig. S3.

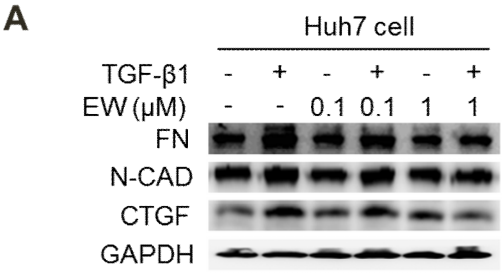

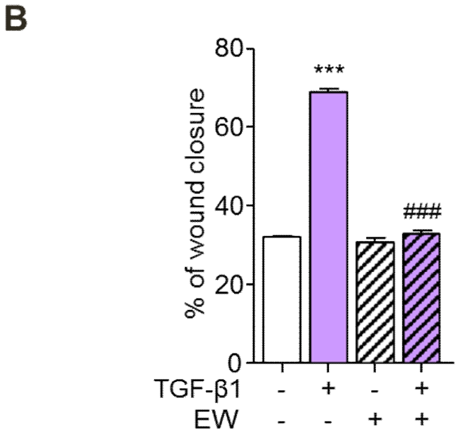

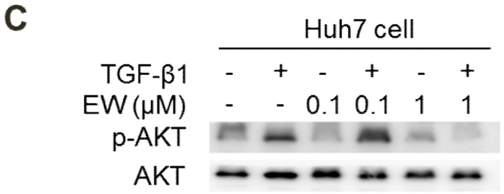

(A) Effects of EW-7197 on the protein expression levels of fibronectin (FN), N-cadherin (N-CAD), and CTGF in Huh7 cells. Cells were treated with the indicated concentration of EW-7197 in the presence or absence of TGF-β1 (2 ng/ml) for 24 h. GAPDH was used as a reference. (B) Effects of EW-7197 (1 μM) on the migration of HepG2 cells. Cells were treated with EW-7197 in the presence or absence of TGF-β1 (2 ng/ml) for 30 h. (C) Effects of EW-7197 on AKT phosphorylation in Huh7 cells. Cells were treated with the indicated concentration of EW-7197 in the presence or absence of TGF-β1 (2 ng/ml) for 24 h. AKT was used as a reference.

Supplementary Fig. S4.

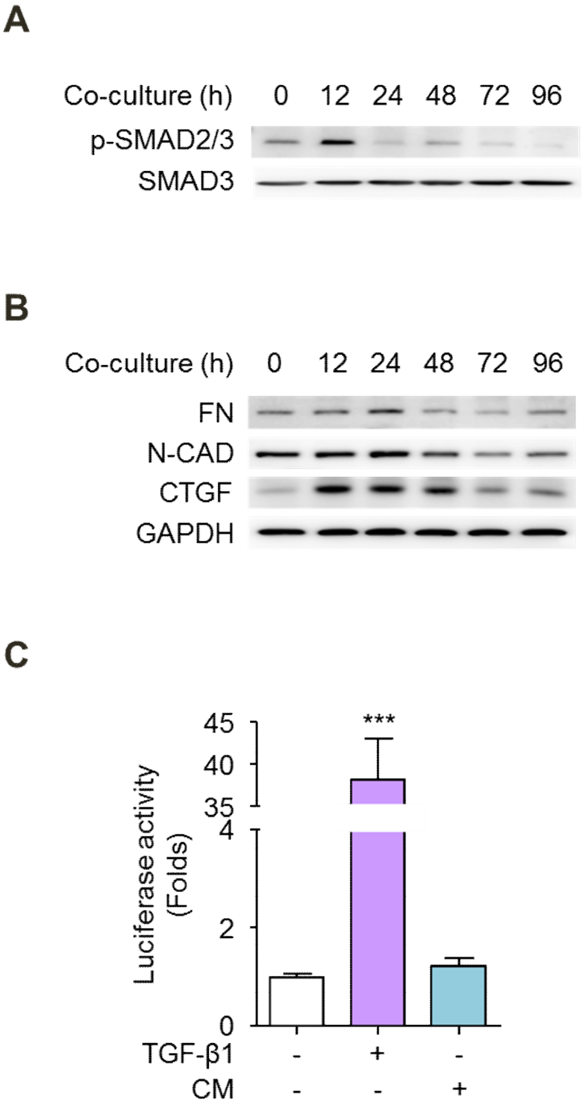

(A) Effects of co-culture with activated LX-2 cells on Smad2/3 phosphorylation in SK-HEP1 cells. Smad3 was used as a reference. (B) Effects of co-culture with activated LX-2 cells on the protein expression levels of fibronectin (FN), N-cadherin (N-CAD), and CTGF in SK-HEP1 cells. GAPDH was used as a reference. (C) Effects of CM on 3TP-Lux promoter activity in SK-HEP1 3TP-Lux stable cells. Cells were treated with TGF-β1 (2 ng/ml) or CM for 24 h. TGF-β1-treated cells were used as a positive control. $^{***}p<0.001$ vs. untreated control.

Supplementary Fig. S5.

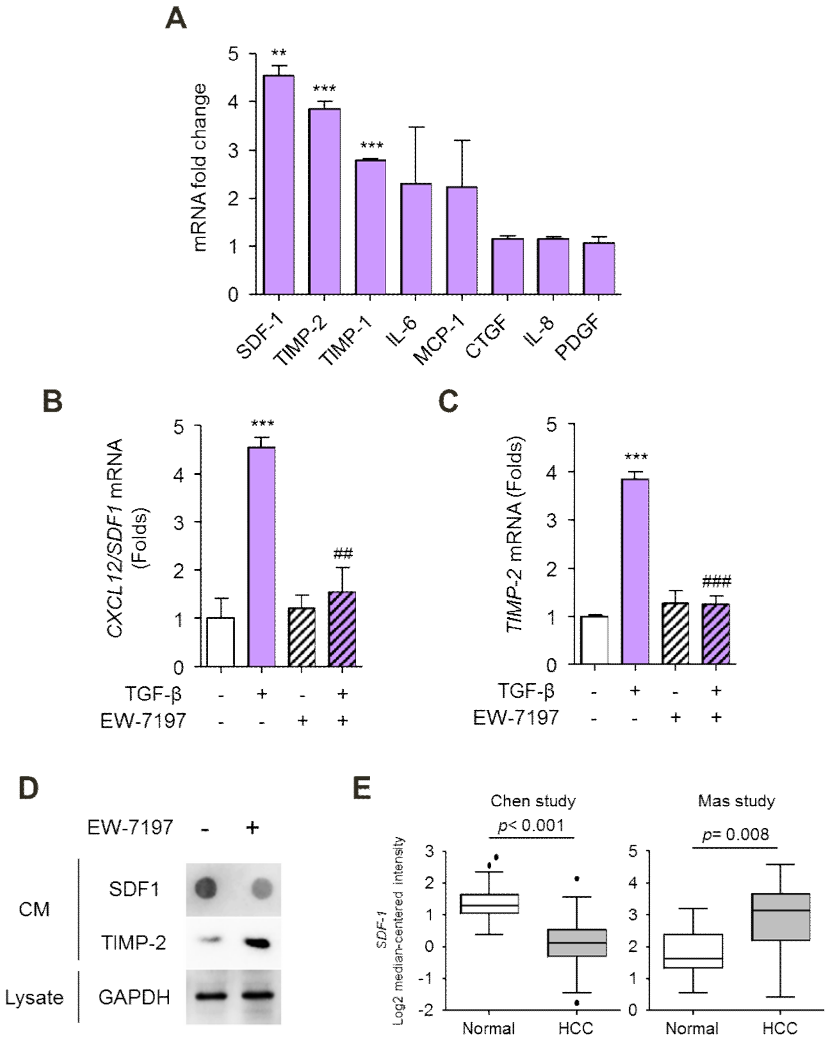

(A) mRNA expression levels of secreted factors in TGF-β-activated LX-2 cells. Cells were treated with TGF-β1 (2 ng/ml) for 24 h. HPRT was used as a reference. The results were expressed as fold change relative to untreated control. (B) mRNA expression levels of SDF-1 in LX-2 cells. Cells were treated with EW-7197 (1 μM) in the presence or absence of TGF-β1 (2 ng/ml) for 24 h. HPRT was used as a reference. (C) mRNA expression levels of TIMP-2 in LX-2 cells. Cells were treated with EW-7197 (1 μM) in the presence or absence of TGF-β1 (2 ng/ml) for 24 h. HPRT was used as a reference. (D) Effects of EW-7197 on SDF-1 and TIMP-2 expression in CM. The GADPH level in an aliquot of total cell lysate was used as a reference. (E) mRNA expression levels of SDF-1 in HCC patients from the Chen and Mas dataset from Oncomine (www.oncomine.com). $^{**}p<0.01$ vs. untreated control, $^{***}p<0.001$ vs. untreated control, $^{\#\#}p<0.01$ vs. TGF-β1-treated control, and $^{\#\#\#}p<0.001$ vs. TGF-β1-treated control.

Supplementary Fig. S6.

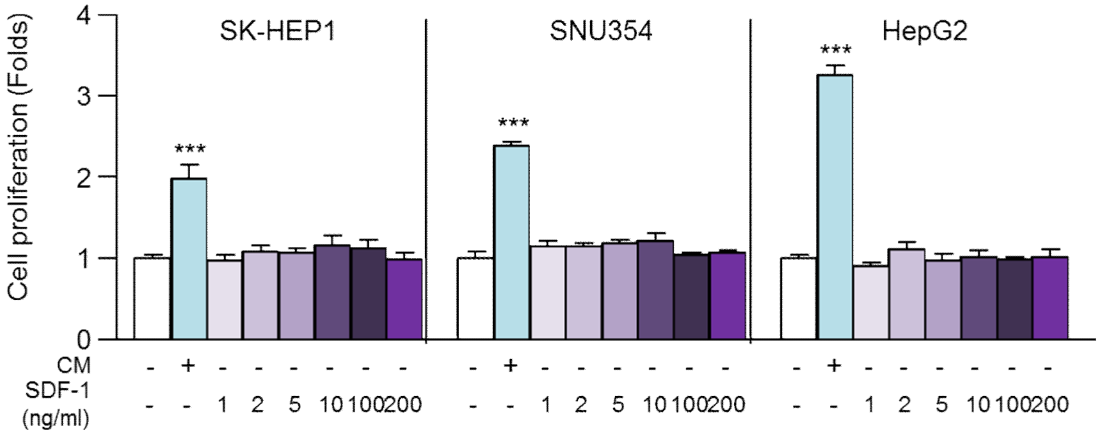

Effects of SDF-1 on the proliferation of SK-HEP1, SNU354, and HepG2 cells. Cells were treated with CM or the indicated concentration of SDF-1 for 72 h. $^{***}p<0.001$ vs. untreated control.

Supplementary Fig. S7.

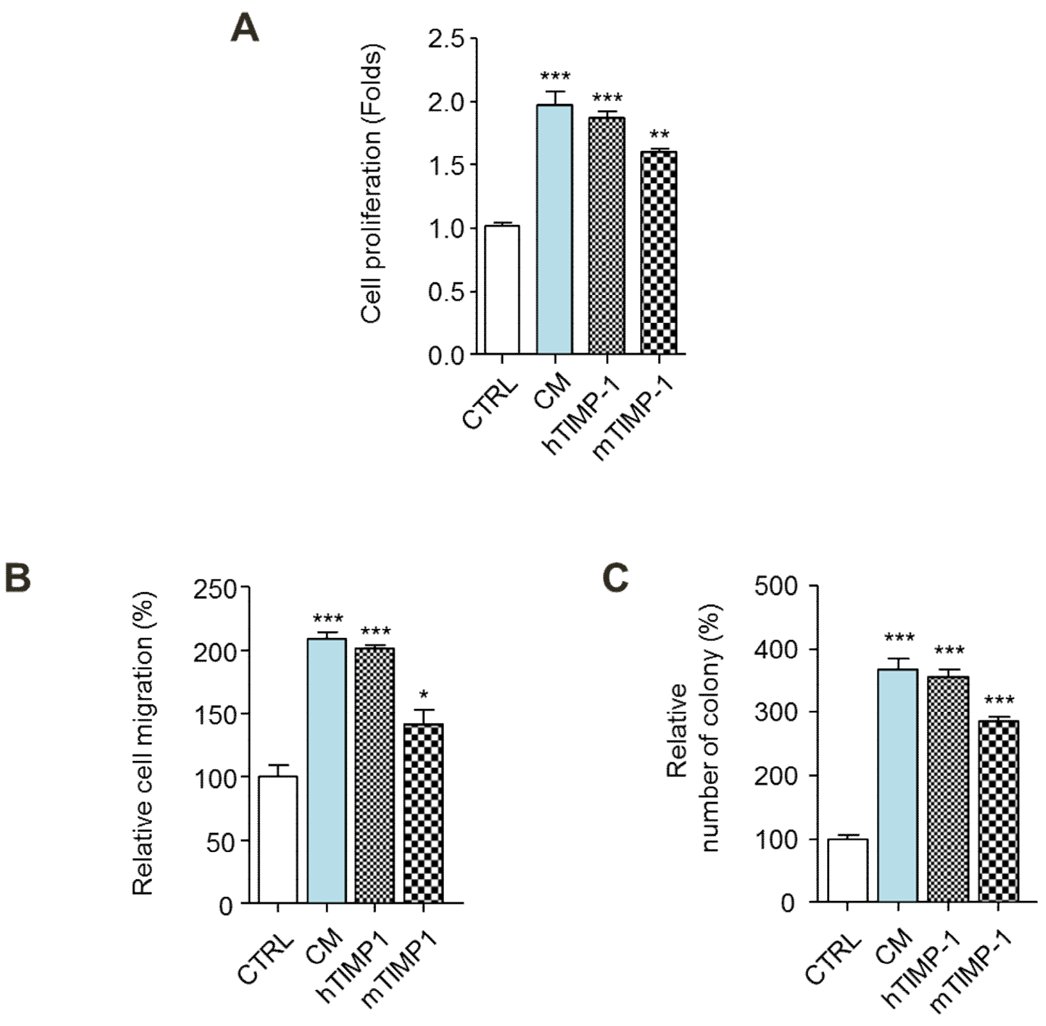

(A) Effects of recombinant TIMP-1 on the proliferation of SK-HEP1 cells. Cells were treated with CM or recombinant TIMP-1 (250 ng/ml) for 72 h. (B) Densitometric analysis of wound-healing assay of SK-HEP1 cells. Cells were treated with CM or recombinant TIMP-1 (250 ng/ml) for 30 h. (C) Densitometric analysis of soft agar assay of SK-HEP1 cells. Cells were treated with CM or recombinant TIMP-1 (250 ng/ml) for 24 h. After treatment, the cells were counted and placed in soft agar for colony assay to determine cell survival. $^{*}p<0.05$ vs. untreated control, $^{**}p<0.01$ vs. untreated control, and $^{***}p<0.001$ vs. untreated control.

Supplementary Fig. S8.

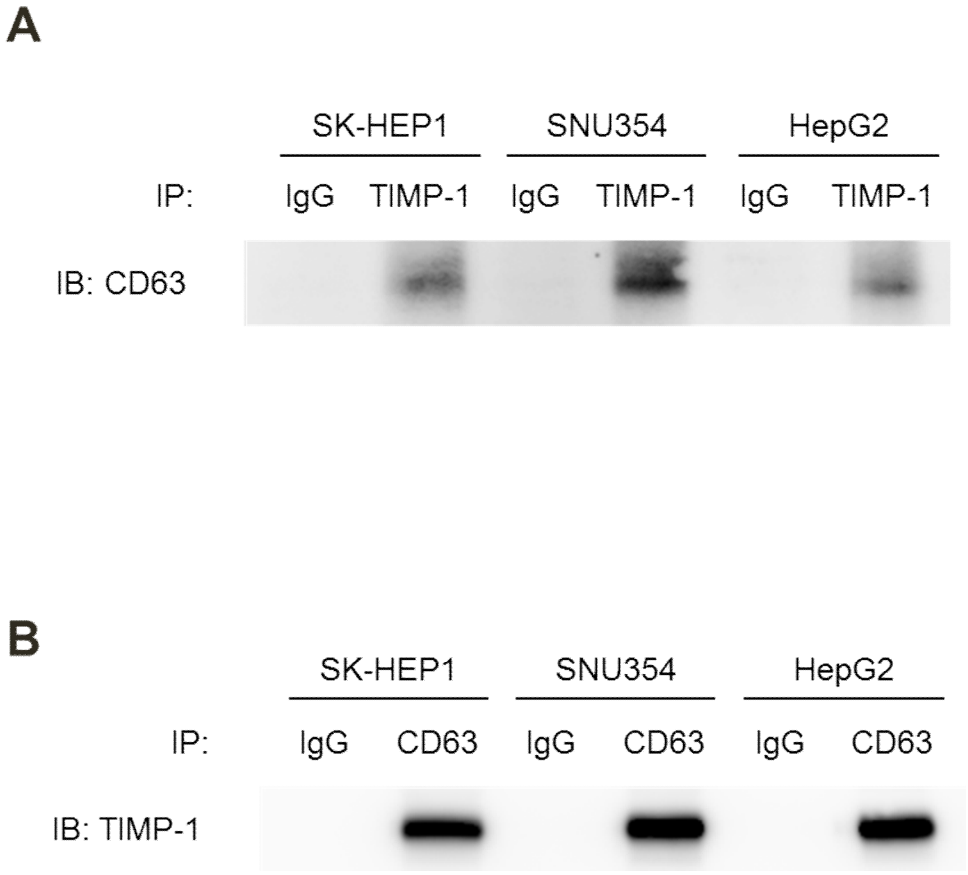

(A) Anti-TIMP-1 immunoprecipitates of CM-treated SK-HEP1, SNU354, and HepG2 cell lysates were analyzed by Western blot analysis with anti-CD63 antibody. (B) Anti-CD63 immunoprecipitates of CM-treated SK-HEP1, SNU354, and HepG2 cell lysates were analyzed by Western blot analysis with anti-TIMP-1 antibody. Cells were treated with CM for 24 h.

Supplementary Fig. S9.

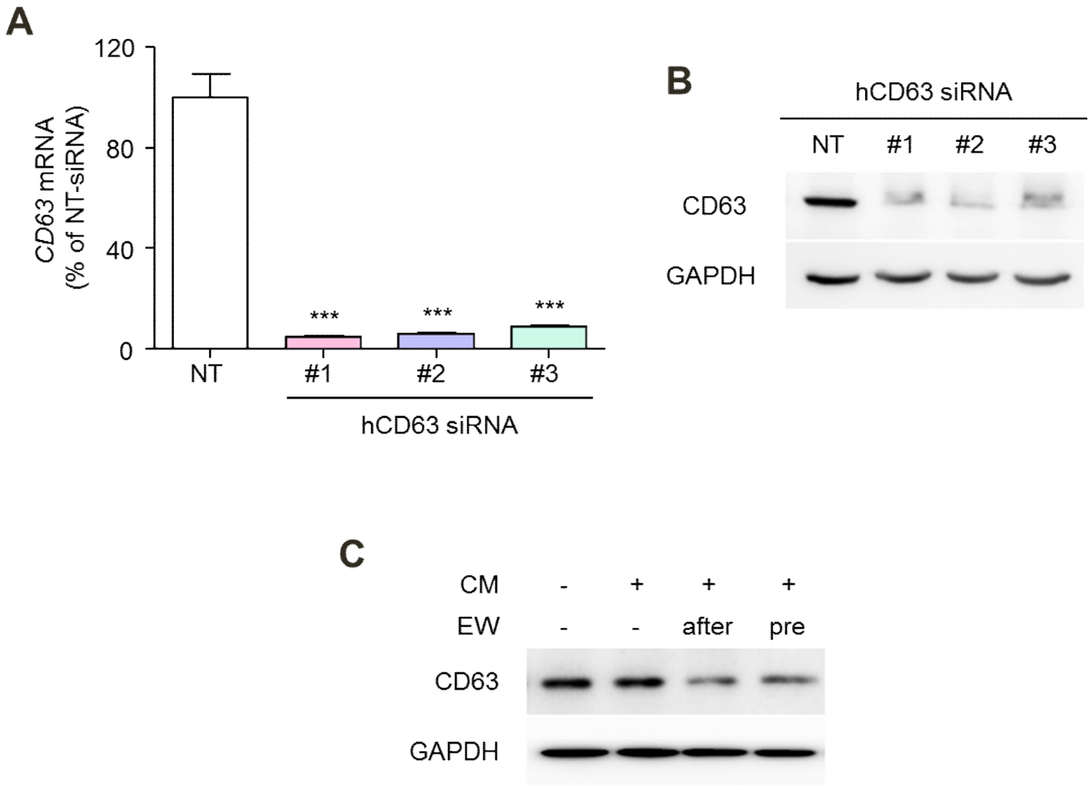

(A, B) CD63 mRNA and protein were silenced by the transfection of CD63 siRNA into SK-HEP1 cells. After transfection, CD63 mRNA (A) and protein (B) expression was evaluated. HPRT and GAPDH expression levels were used as references. $^{***}p<0.001$ vs. NT-siRNA transfected cells. (C) Effects of EW-7197 on the protein expression levels of CD63 in SK-HEP1 cells. Cells were treated with CM in the presence or absence of EW-7197 (1 μM) for 24 h. GAPDH was used as a reference.

Supplementary Fig. S10.

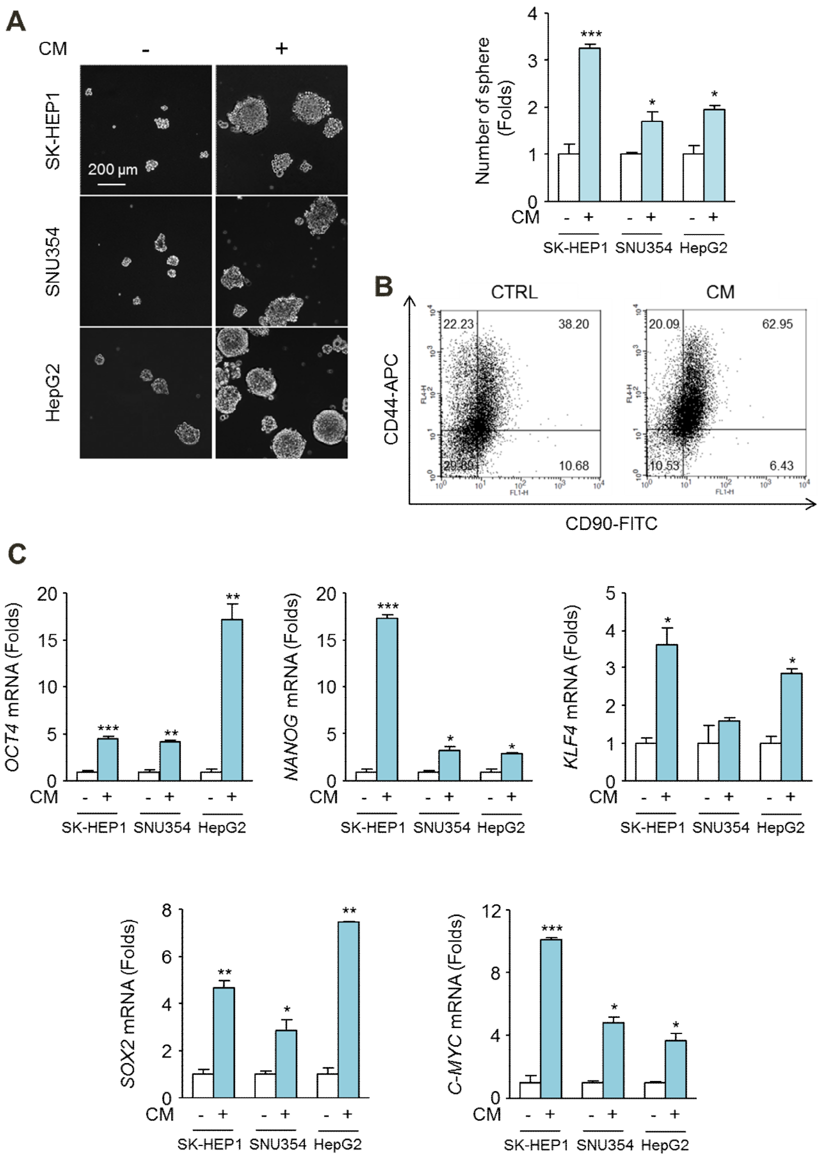

(A) Effects of CM on the sphere-forming abilities of HCC cell lines. Representative images (left) and the quantification of secondary spheres formed in self-renewal assay (right) of SK-HEP1, SNU354, and HepG2 cells. Scale bars: 200 μm. (B) Flow cytometric analysis of CD44 and CD90 in SK-HEP1 cells. Cells were treated with CM for 24 h. (C) mRNA expression levels of OCT4, NANOG, KLF4, SOX2, and c-MYC in SK-HEP1, SNU354, and HepG2 cells. Cells were treated with CM for 24 h. HPRT was used as a reference. $^{*}p$<0.05 vs. untreated control, $^{**}p$<0.01 vs. untreated control, and $^{***}p$<0.001 vs. untreated control.

Supplementary Fig. S11.

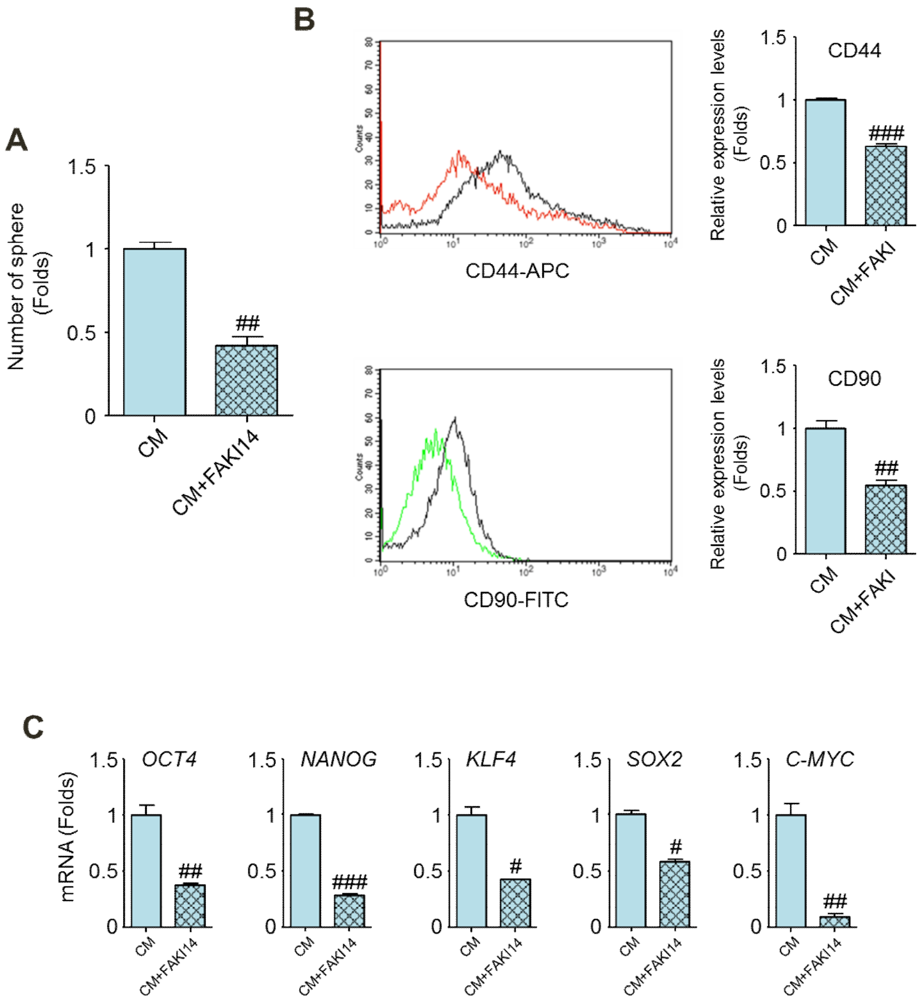

(A) Effects of FAK inhibitor-14 on the sphere-forming abilities of SK-HEP1 cells. (B) Effects of FAK inhibitor-14 on CD44 (up) and CD90 (down) expression in SK-HEP1 cells. Cells were treated with CM in the presence or absence of EW-7197 (1 μM) for 24 h. Black histograms: CM-treated cells; red (CD44-APC) and green (CD90-FITC) histograms: combination of CM- and FAKI-14-treated cells. (C) Effects of FAK inhibitor-14 on the mRNA expression levels of OCT4, NANOG, KLF4, SOX2, and c-MYC in SK-HEP1 cells. Cells were treated with CM in the presence or absence of EW-7197 (1 μM) for 24 h. HPRT was used as a reference. $^{\#}p<0.05$ vs. CM-treated control, $^{\#\#}p<0.01$ vs. CM-treated control, and $^{\#\#\#}p<0.001$ vs. CM-treated control.

Supplementary Tables

Supplementary table S1. siRNA sequence

	Sense	Antisense
Human CD63		
#1	CUCUGUUGCCAGUGGUCAU	AUGACCACUGGCAACAGAG
#2	GAUGGAGAAUUACCCGAAA	UUUCGGGUAAUUCUCCAUC
#3	CUGUUACCGCGUCACAUGA	UCAUGUGACGCGGUAACAG

Supplementary table S2. Antibodies for western blot, immunofluorescence, immunoprecipitation, and IHC.

Antibody	Company	Product number
p-Akt (ser473)	Cell signaling	4060
Akt	Cell signaling	9272
α-SMA	Sigma	A2547
CD63	Santa Cruz Biotechnology	sc-15363
CTGF	Santa Cruz Biotechnology	sc-14939
Fibronectin	BD biosciences	610077
p-FAK	Santa Cruz Biotechnology	sc-11765-R
FAK	Millipore	05-537
GAPDH	Cell signaling	5174
N-cadherin	BD biosciences	610921
SDF-1	Cell signaling	3530
p-Smad2/3	Santa Cruz Biotechnology	sc-11769
Smad3	Ab frontier	AF9F7
TIMP-1	Santa Cruz Biotechnology	sc-5538
TIMP-2	Abcam	ab1828

Supplementary table S3. Primers for qRT-PCR.

	Forward	Reverse
Human		
c-MYC	CTTCTCTCCGTCCTCGGATTCT	GAAGGTGATCCAGACTCTGACCTT
CTGF	GGTTACCAATGACAACGCCT	TGCTCCTAAAGCCACACCTT
CXCL12/SDF1	GCTTTGAGTGACTGGGTT	GTGGCAAGATGATGGTTT
HPRT	CTTTGCTTTCCTTGGTCAGG	GGTCCTTTTCACCAGCAAGC
IL-6	GCCGCCCCACACAGACA	CCGTCGAGGATGTACCGAAT
IL-8	GTGCAGTTTTGCCAAGGAGT	CTCTGCACCCAGTTTTCCTT
KLF4	CAAGTCCCGCCGCTCCATTACCAAGAG	CCACTGTCTGGGATTTAAAAATGCCTC
MCP-1	ATGCCCCAGTCACCTGCTGT	GTTCAAGTCTTCGGAGTTTGG
NANOG	CAGCTGTGTGTACTCAATGATAGATTT	ACACCATTGCTATTCTTCGGCCAGTTG
OCT4	ACATCAAAGCTCTGCAGAAAGAACT	CTGAATACCTTCCCAAATAGAACCC
PDGF	CCCCTGCCCATTCGGAGGAAGAGA	TTGGCCACCTTGACGCTGCGGTG
SOX2	AAATGGGAGGGGTGCAAAAGAGGAG	CAGCTGTCATTTGCTCTGGGTGATG
TIMP-1	GGGCTTCACCAAGACCTACA	TGCAGGGGATGGTAAACA
TIMP-2	ACCCTCTGTGACTTCATCGTGC	GGAGATGTAGCACGGGATCATG

Supplementary methods

Animals, Treatments and Specimen Collection

Female BALB/c-nu/nu mice were purchased from Central Lab Animal, Inc. (Seoul, Korea). The mice were housed with five to seven animals per cage at room temperature under a 12-h light/dark cycle. A normal chow diet and water were provided ad libitum. All experimental procedures were approved by the Animal Care Committee of Ewha Womans University and complied with the NIH Guide for the Care and Use of Laboratory Animals (Institute of Laboratory Animal Resources, National Research Council, WA, USA). At 1 h after the last EW-7197 administration, the animals were deeply anaesthetized by intraperitoneal injection of Zoletil/Rompun (2:1 mixture). The animals were exsanguinated via the axillary artery, followed by the collection of sera and excision of the liver, spleen, kidneys, and lungs. The sera and tissues were immediately stored at -80°C until use.

Peripheral Blood Mononuclear Cell (PBMC) Purification

White blood cells (WBCs) were isolated from mouse peripheral blood by the centrifugation of whole blood at 1,000 g at 4°C for 15 min to form a buffy coat. WBCs separated from the buffy coat were fixed, while contaminated red blood cells (RBCs) were lysed with BD FACS Lysing^{TM} Solution (BD Biosciences, San Jose, CA, USA). WBCs were smeared on silane-coated micro slides (Muto Pure Chemicals, Tokyo, Japan). The p-Smad2/3 protein level was visualized by immunofluorescence.

Luciferase Reporter Gene Assay

SK-HEP1 3TP-Lux stable cells were seeded into a 96-well plates and were treated with

various concentrations of activin receptor-like kinase 5 (ALK5) inhibitors in the presence or absence of TGF-β1 (2 ng/ml) for 24 h. Luminescence was measured in cell lysates with a luminometer (Micro Lumat Plus; Berthold Technologies Berthold Technologies, Bad Wildbad, Germany).

Co-culture of HSCs with HCC cells

TGF-β-activated LX-2 cells (5×10^4 cells per insert) were seeded into upper inserts of Transwell cell culture chambers (6 wells, 0.4-μm pore size; Falcon, Oxnard, CA) and co-cultured with SK-HEP1 cells (5×10^5 cells per well) in the lower chambers without direct contact. SK-HEP1 cells alone were cultured in a 6-well plate as controls. After co-culture of LX-2 cells with SK-HEP1 cells, upper inserts containing LX-2 cells were discarded. SK-HEP1 cells co-cultured with or without LX-2 cells were washed and then used for subsequent experiments.

Self-Renewal Assay

For the assessment of sphere-forming efficiency, trypsinized cells (8 cells/μl) were seeded in 6-well ultra-low attachment plates (Corning Science Products, Corning, NY, USA) and grown in CM. After an additional 7 days, primary spheres were dissociated into single cells and re-plated in B27-supplemented DMEM-F12 media. At least three repeats of both the primary and secondary sphere assays were performed for each cell line. Sphere size and number were measured at 7 days after the cells were seeded.

Flow Cytometric Analysis

Cells were trypsinized and stained with CD44-APC and CD90-FITC for 30 min at 4°C in the dark. The stained cells were washed twice with PBS and analyzed by FACS (Becton Dickinson, Mountain View, CA, USA). Unstained cells and those stained with CD44 or CD90 alone served as controls.
